# The Universal Role of Gallium in Promoting Methanol Formation across CO_2_ Hydrogenation Catalysts

**DOI:** 10.1021/acs.accounts.5c00581

**Published:** 2025-11-06

**Authors:** Colin Hansen, Wei Zhou, Christophe Copéret

**Affiliations:** Department of Chemistry and Applied Biosciences, 27219 ETH Zürich, Vladimir Prelog Weg 1-5, CH-8093 Zurich, Switzerland

## Abstract

The production of value-added chemicals from CO_2_ has been a thriving topic of research for the past few decades because of its contribution to a circular carbon economy. Combined with CO_2_ capture and storage, thermocatalytic hydrogenation of CO_2_ to CH_3_OH with green or blue hydrogen, offers an attractive route to mitigate CO_2_ emissions and to decarbonize the chemical industry. Numerous studies have been focused on catalysts based on supported metallic nanoparticles; these catalysts consist of at least one transition or coinage metal and a promoter element combined with an oxide support to disperse the active phase. Besides Zn-promoters used in Cu-based hydrogenation catalysts, numerous reports point to Ga as a promoter for methanol synthesis. In recent years, Ga has been shown to convert almost all transition metals toward selective methanol synthesis, but its specific role remains a topic of discussions.

In this Account, we summarize how surface organometallic chemistry (SOMC) has enabled the discovery of novel catalysts and the development of detailed structure–activity relationships. Particularly, we show that Ga uniquely generates alloys with transition and coinage (Cu) metal elements across groups 8–11 and converts them into selective methanol synthesis catalysts. Specifically, we highlight the role of M–Ga alloy formation, alloy stability, and the formation of M­(Ga)–GaO_*x*_ interfaces under reaction conditions. This has been possible thanks to the combination of SOMC, which enables the formation of supported nanoparticles with tailored compositions and interfaces, and state-of-the-art characterization including *operando* techniques along with computational modeling, including *ab initio* molecular dynamic calculations. Dynamic alloying–dealloying behaviors under reaction conditions and the formation of M/MGa–GaO_*x*_ interfaces are identified as key drivers for efficient methanol formation.

## Graphical abstract

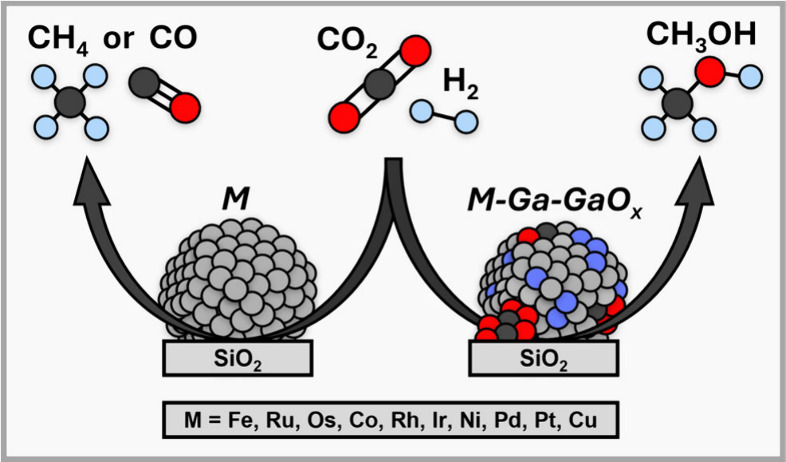

## Key References

-

Lam, E. ; Noh, G. ; Chan, K. W. ; Larmier, K. ; Lebedev, D. ; Searles, K. ; Wolf, P. ; Safonova, O. V. ; Copéret, C. Enhanced CH_3_OH selectivity in CO_2_ hydrogenation using Cu-based catalysts generated via SOMC from GaIII single-sites. Chem. Sci. 2020, 11, 7593–7598 34094136 10.1039/d0sc00465kPMC8159433.^1^ *We demonstrated how Surface Organometallic Chemistry can be exploited to produce well-defined Cu–Ga bimetallic catalysts, in which the methanol formation mechanism could be studied in detail.*
-

Docherty, S. R. ; Phongprueksathat, N. ; Lam, E. ; Noh, G. ; Safonova, O. V. ; Urakawa, A. ; Copéret, C. Silica-Supported PdGa Nanoparticles: Metal Synergy for Highly Active and Selective CO_2_-to-CH_3_OH Hydrogenation. JACS Au 2021, 1, 450–458 34467307 10.1021/jacsau.1c00021PMC8395611.^2^ *A material with significantly higher performance, PdGa@SiO*_*2*_ *, was reported, and in accordance with the Cu–Ga systems, an efficient alloying–dealloying behavior of the catalyst could be observed.*
-

Zhou, W. ; Brack, E. ; Ehinger, C. ; Paterson, J. ; Southouse, J. ; Coperet, C. Reactivity Switch of Platinum with Gallium: From Reverse Water Gas Shift to Methanol Synthesis. J. Am. Chem. Soc. 2024, 146, 10806–10811 38572914 10.1021/jacs.4c01144.^3^ *The reactivity of platinum-based catalysts, which are efficient reverse water–gas shift catalysts, was switched to methanol formation in high efficiency by the addition of Ga. The inhibiting role of Ga* ^*III*^ *single sites was revealed, by a tailored support study.*
-

Zhou, W. ; Hansen, C. ; Cao, W. ; Brack, E. ; Docherty, S. R. ; Ehinger, C. ; Wang, Y. ; Wang, C. ; Copéret, C. Gallium: A Universal Promoter Switching CO_2_ Methanation Catalysts to Produce Methanol. JACS Au 2025, 5, 217–224 39886588 10.1021/jacsau.4c00893PMC11775694.^4^ *We expanded the library of M–Ga alloys to classical methanation type metals. We demonstrated the formation of highly stable M–Ga alloys, which suppress the methanation activity, while stabilizing methoxy species on the way to methanol.*

## 1 Introduction

The growing anthropogenic carbon emissions over the past few decades have raised undoubtful environmental concerns, promoting an urgent search for sustainable chemical solutions. Carbon capture and utilization (CCU)^5–7^ has gained significant attention for transforming CO_2_, a major greenhouse gas, into value-added chemicals, thereby contributing to a circular carbon economy and promoting more efficient use of resources.^8–11^ The conversion of CO_2_ to methanol has emerged as a promising avenue to mitigate CO_2_ emissions. This transformation not only offers a viable pathway to mitigate carbon emissions but also serves as a cornerstone for sustainable chemical manufacturing. The current production of methanol (100 Mt/a) involving classical reforming technologies and syngas (H_2_/CO) conversion emits ca. +1 kg_CO_2__/kg_MeOH_, while moving to the hydrogenation of CO_2_ with green H_2_ would consume ca. −1 kg_CO_2__/kg_MeOH_. Thus, the envisioned “methanol economy” relies on converting captured CO_2_ and renewably sourced hydrogen (e.g., green or blue H_2_) into methanol over a heterogeneous catalyst. This approach represents a practical step toward a CO_2_-neutral future by integrating carbon recycling into the energy and chemical sectors (Figure 1a).^12–16^

**Figure 1:**
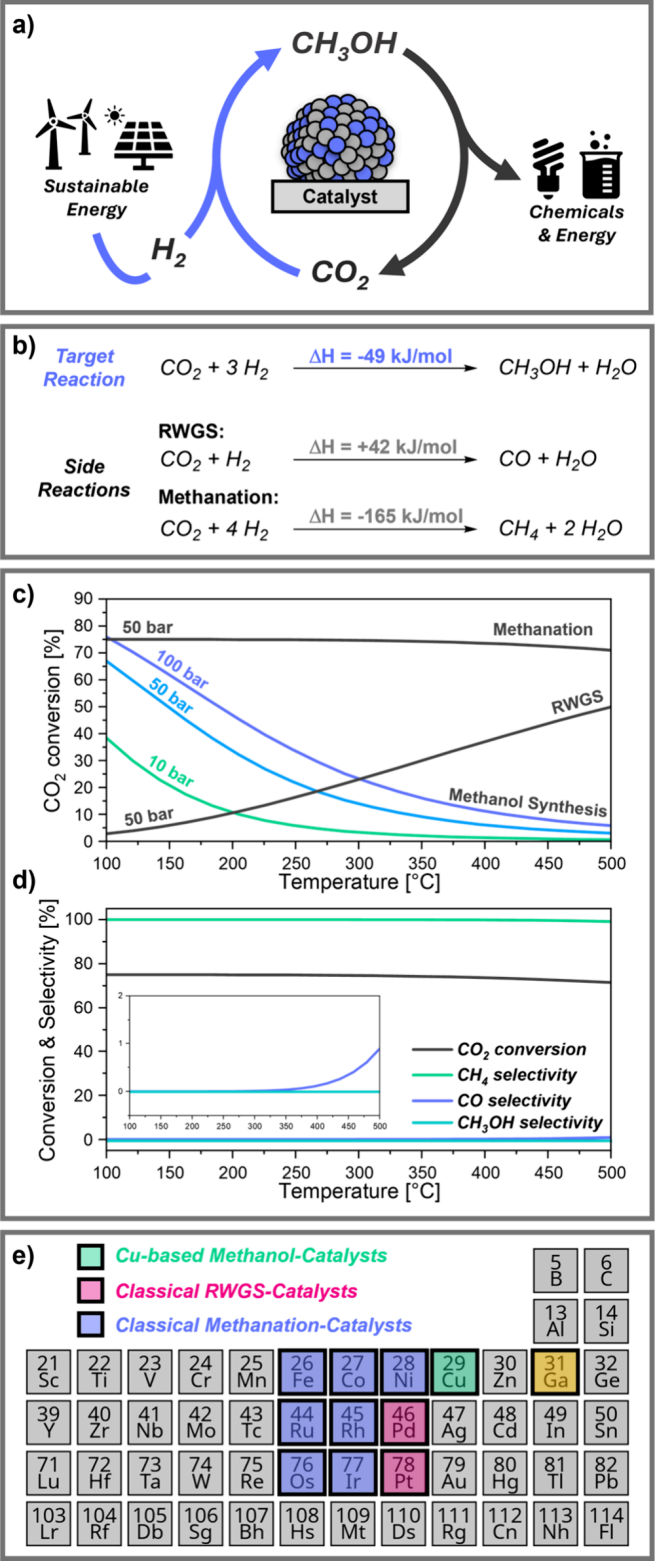
(a) Circular methanol economy; (b) Side reactions in CO_2_-hydrogenation; (c) Thermodynamic CO_2_ conversion for the three main reactions in a 1:3:1 CO_2_/H_2_/Ar mix; (d) Conversion and selectivities vs temperature at 50 bar; (e) Periodic table section showing catalytic metals, classified by reduced-state, monometallic CO_2_-hydrogenation behavior on SiO_2_.

Methanol synthesis from CO_2_-hydrogenation is exothermic and reduces the number of gas molecules (Figure 1b). Thus, high pressures and low temperatures favor CO_2_ conversion (Figure 1c). However, due to CO_2_ inertness and the need for high reaction rates, temperatures above 200 °C are typically required.

Under such conditions, the reverse water–gas shift (RWGS) reaction becomes thermodynamically favored, while methanation remains both thermodynamically and kinetically favorable, limiting methanol selectivity. As Figure 1d shows, at 230 °C and 50 bar, methane selectivity approaches 100% when CH_3_OH, CH_4_, and CO are possible products. The suppression of methanation can already drastically increase the conversion to methanol when RWGS is controlled (Figure S2). These side reactions reduce the methanol yield and waste hydrogen, making catalysts designed with high activity, selectivity, and stability while suppressing undesired reactions a key challenge.

Because of the similarity between syngas-to-methanol and CO_2_-based methanol synthesis, Cu-based catalysts have been widely studied.^17,18^ Research has focused on identifying promoters to improve performance, where Zn, a key element of industrial syngas catalysts (Cu/ZnO/Al_2_O_3_), is a well-known promoter that greatly enhances methanol selectivity. Ga has also been reported to enhance methanol selectivity, sometimes surpassing Zn, though the roles of Ga and Zn promoters remain debated.^19–22^

Besides Cu, late transition metals (groups 8–10) are active in CO_2_-hydrogentation but usually catalyze RWGS (Pd, Pt) or methanation (Fe, Co, Ni, Ru, Rh, Os, Ir) in their metallic form (Figure 1e). As with Cu, the presence of Zn or Ga as promoter or in supports can alter their reactivity, enhancing activity or shifting selectivity. As will be discussed, adding Ga causes most transition metals, except Fe and Co, to favor methanol synthesis. This universal promotional effect, central to this perspective, demands a deeper understanding of the factors driving such reactivity patterns.

A key barrier to molecular-level understanding is that catalysts made by “classical” methods (precipitation, impregnation) form complex materials.^23^ These seemingly simple, industrially relevant methods involve dissolution and precipitation in aqueous solution, producing ill-defined systems (Figure 2a). Such complexity hinders molecular-level characterization and makes identification of structure–reactivity relationships difficult by comparison with their homogeneous counterparts. Surface organometallic chemistry (SOMC) has emerged as a powerful method to create tailored catalysts for studying the role(s) of promoters.^24,25^ It uses thermally treated supports to control reactive surface moieties, typically OH groups.^24,26–28^ When performed in dry organic solvents, SOMC enables controlled metal deposition, where organometallic precursors react with support functionalities to generate well-defined surface metal sites, avoiding dissolution/precipitation and complex interfaces.^29,30^ Subsequent thermal treatment under a reductive atmosphere (e.g., H_2_) allows for the generation of supported nanoparticles with tailored interfaces or compositions (Figure 2b), suitable for detailed characterization. Combined catalytic studies and *in situ*/*operando* spectroscopy enable molecular-level structure–activity relationships and guidelines for rational catalyst design.

**Figure 2:**
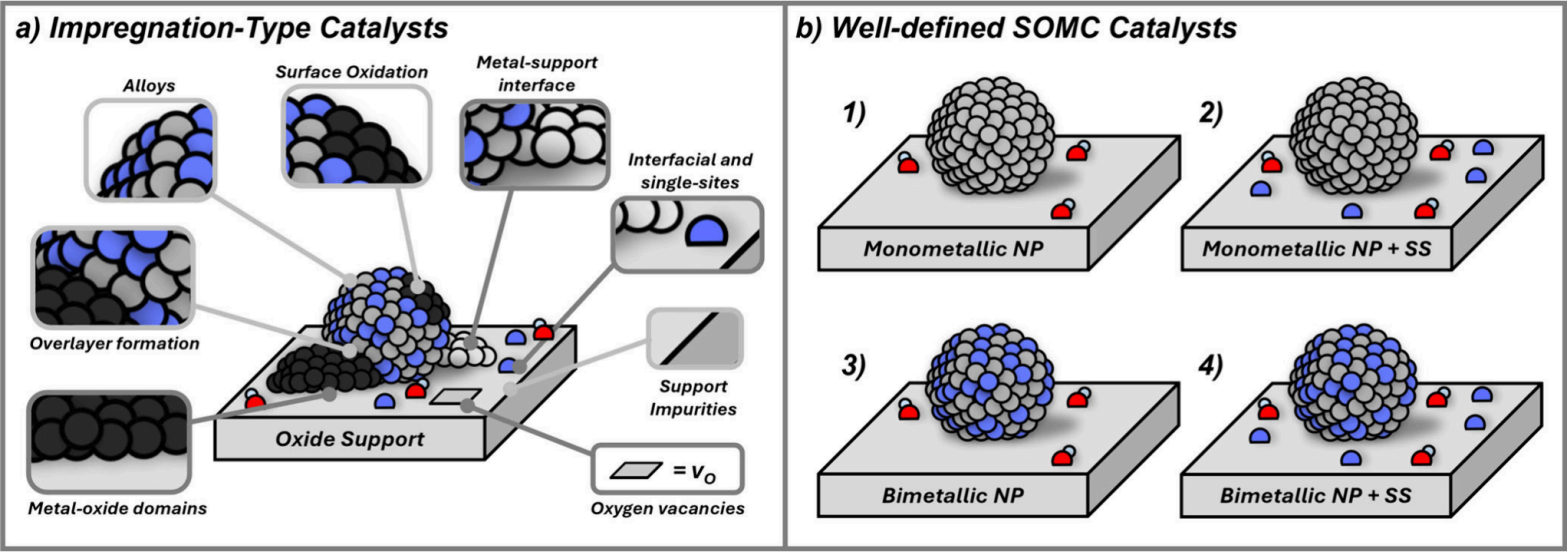
(a) A range of support and dopant effects on heterogeneous catalysts, which lead to the complexity of classically investigated materials (adapted from Docherty et al.^37^); (b) A range of well-defined material compositions which can be achieved by Surface organometallic chemistry approaches. (NP = Nanoparticle, SS = Single-sites).

This Account discusses recent advances in SOMC, especially strategies to create bimetallic supported catalysts (Section 3), emphasizing the universal promotional role of Ga in methanol synthesis under CO_2_-hydrogenation (Figure 1e). We present results for Ga-promoted catalytic systems based on Cu (Section 4); Pd and Pt, known in RWGS (Section 5); and Fe, Co, Ni, Ru, Rh, Os, and Ir best known for their methanation activities (Section 6). We highlight the role of Ga as part of MGa alloys and Ga^III^ Lewis acid sites in methanol formation, catalyst evolution, and intermediate identification via combined *in situ*/*operando* spectroscopy and computations.

## 2 Preparing M–Ga Model Catalysts by SOMC

Gallium shows significant activity or promotional effects in catalytic processes, notably alkane dehydrogenation and CO_*x*_-hydrogenation.^31–33^ Ga silicates readily activate H_2_ and alkanes and are known to catalyze alkane dehydrogenation as well as aromatization. Silica-supported Ga^III^ single sites have thus been first prepared via SOMC to study the intrinsic activity of Ga in propane dehydrogenation (PDH) and then used to investigate the related bimetallic PtGa PDH catalysts.^34–36^

In this context, Ga^III^ single sites are generated by grafting Ga­(OTBOS)_3_THF (OTBOS = OSi­(O^t^Bu)_3_)_3_) onto partially dehydroxylated silica (SiO_2–700_), followed by high-vacuum thermal treatment to remove TBOS ligands. This yields Ga^III^ sites along the surface −OH groups that can be used for further grafting and forming bimetallic systems. For Ga-promoted Pt, PDH model catalysts are made by grafting (COD)­Pt­(OTBOS)_2_ onto residual OH groups; reduction under H_2_ forms a PtGa alloy with approx. 10% residual Ga sites.

This approach has since been generalized and further improved to study numerous bimetallic systems and catalytic reactions, in particular, MGa bimetallic systems in the context of CO_2_ hydrogenation as discussed below.

The *single-site approach* (Method A) directly derived from the preparation of the silica-supported PtGa systems has been generalized (Figure 3a and Table S1), as Ga^III^ surface sites (generated from the grafting of Ga­(OTBOS)_3_THF, a thermolytic molecular precursor)^35^ are reduced in the presence of most transition or coinage metals (M) to form MGa alloys with some residual Ga^III^ sites.^37^ Bimetallic MGa systems with M = Fe, Co, Ni, Cu, Ru, Rh, Pd, Os, Ir, or Pt have been studied using tailored SOMC molecular precursors (Figure 3c).^4,38–40^

**Figure 3:**
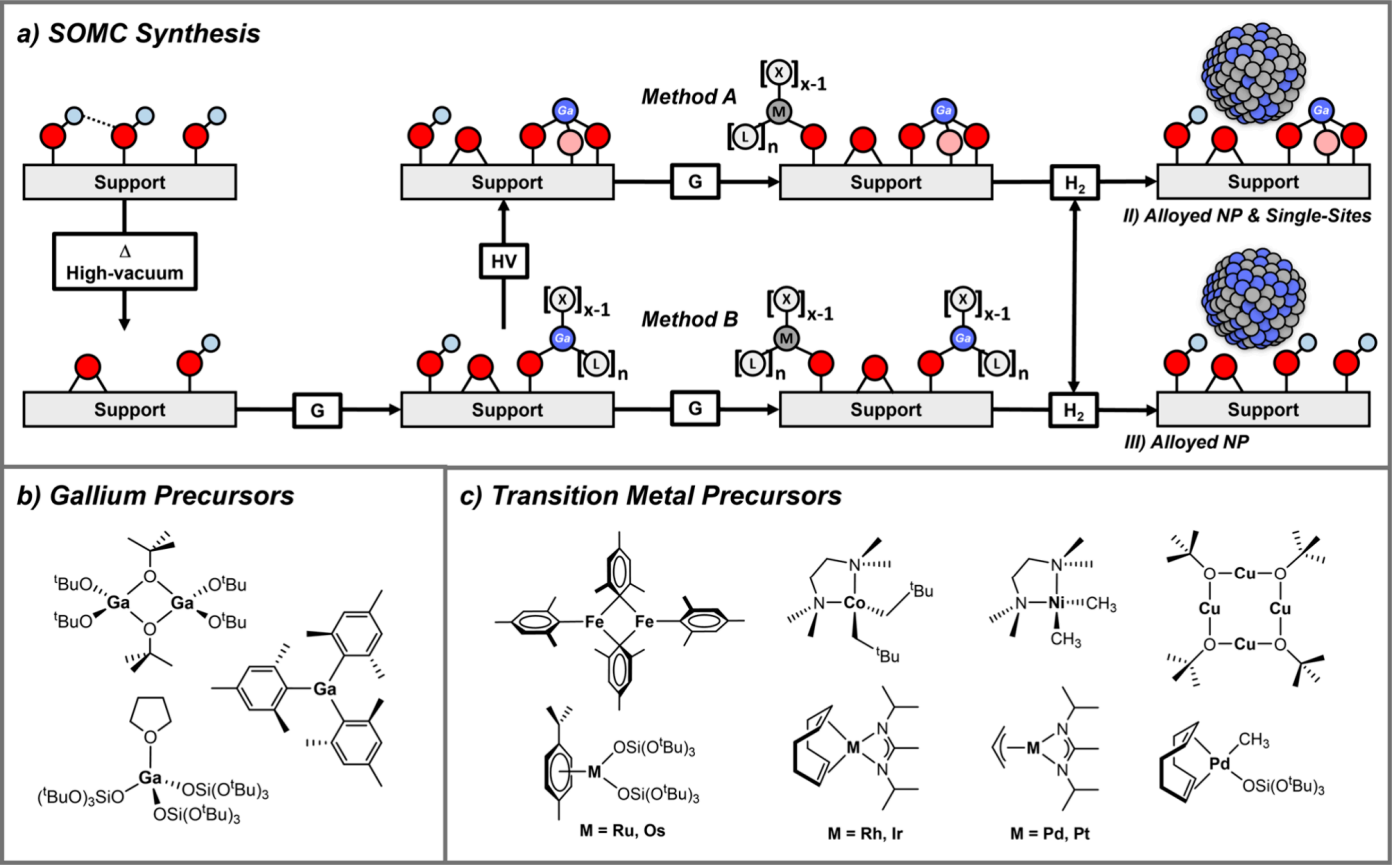
(a) Flow-chart containing the most commonly employed techniques for the formation of mono- and multimetallic supported Ga-promoted catalysts by an SOMC approach (G = grafting, PT = post-treatment); (b) SOMC precursors for the installation of Ga; (c) Tailored transition-metal precursors used for synthesizing well-defined SOMC materials.

For instance, Cu–Ga bimetallic catalysts were synthesized from Ga^III^ single sites using [Cu_4_(O^t^Bu)_4_] as the Cu-source, yielding nanoparticles with a size of 4.6 ± 1.4 nm.^1^ Other precursors like [Cu_4_(Mes)_4_] (Mes = mesityl) can also be used.^41^ As discussed above, silica-supported PtGa (1.9 ± 0.8 nm) nanoparticles are prepared using either (COD)­Pt­(OTBOS)_2_ or Pt­(All)­(DIA) (COD = cyclooctadiene, All = allyl, DIA = *N*,*N*′-diisopropylacetimidinate) as convenient precursors. Notably, the corresponding carbon-supported PtGa catalyst can also be prepared but requires *sequential grafting* of GaMes_3_ and Pt­(All)­(DIA) (Method B), yielding nanoparticles with a size of 1.4 ± 0.5 nm (*vide infra*).^3^ For Ni, a series of catalysts with various compositions are accessible by tuning the amount of grafted [Ni­(CH_3_)_2_(tmeda)] per surface Ga site (Ni content at ca. 2 wt %, Ni_*x*_Ga_(100–*x*)_@SiO_2_, *x* = 100, 75, 70, 65) with a particle size of 2.0–2.5 nm.^42^ Small, narrowly distributed alloyed PdGa (1.6 ± 0.4 nm) nanoparticles are obtained using Pd­(COD)­Me­((OSi­(O^*t*^Bu_3_)_3_).^2^ Notably, the method is general and has also been applied to generate silica-supported MGa alloys with a fixed M/Ga ratio of ca. 1:1 and a metal loading of ca. 0.5–0.85 M/nm^2^ with Ru, Os, Rh, and Ir using M­(p-cymene)­(OSi­(O^t^Bu)_3_)_2_ (M = Ru, Os) and M­(COD)­(DIA) (M = Rh, Ir) (Figure 3c).^4^ All materials (MGa@SiO_2_ as well as the corresponding M@SiO_2_) show similar particle sizes of ca. 1.8–3.0 nm. For Fe and Co, Co­(Np)_2_(tmeda) (Np = neopentyl, tmeda = tetramethyl ethylenediamine) and Fe_2_Mes_4_ (Mes = mesityl) precursors produced small Fe- and Co-containing nanoparticles that also formed Ga alloys (Figure S4).^39,40^ The small sizes and the formation of amorphous particles prevent X-ray diffraction studies and obtaining more detailed information about the alloy structures in most materials.

Method A produces MGa alloys with residual Ga^III^ sites after H_2_ treatment; residual Ga^III^ depends on M. Using TBOS precursors inherently forms silica/surface silicates, which may be unsuitable for other supports unless a silicate environment is desired.^43^ To obtain metal nanoparticles free of silicate domains, alternative precursors without TBOS can be used, avoiding silicate formation and thermolytic pretreatment (Figure 3b). For Ga, Ga_2_(O^t^Bu)_6_ and Ga­(R)_3_ [Ga_2_Me_6_ or GaMes_3_] are accessible;^44^ for M, perhydrocarbyl (alkyl, aryl) and more recently amidinate or azetidinate^45^ derivatives are suitable (Figure 3c).^38^

We focus herein on the study of silica-supported systems, as this support is mostly “inert” and allows for studying specifically the effect of Ga across a range of M–Ga alloys (M = Fe, Ru, Os, Co, Rh, Ir, Ni, Pd, Pt, and Cu). To study (Ga-)­promotional effects on various supports, a *sequential grafting approach* (Method B)^46^ avoiding the use of TBOS precursors, that always generate silicates, has been developed: a Ga precursor like GaMes_3_ or Ga_2_(O^t^Bu)_6_,^47^ is first grafted on the surface OH groups, then the metal M is grafted on residual silanols, allowing control of the M/Ga ratio at constant total metal content. H_2_ treatment forms supported bimetallic alloys (Figure 3a). This method facilitates alloy formation and better composition control by varying the precursor amounts. For example, the number of Ga^III^ sites on silica was reduced using *sequential grafting* with GaMes_3_ and [Cu_4_(O^t^Bu)_4_].

## 3 The Effect of Gallium on Cu-Based Catalysts

Cu-based catalysts are well-known for the hydrogenation of CO_2_ to methanol. While Cu alone has modest activity and selectivity, promoters like Zn and Ga greatly enhance both. Earlier works have proposed that Ga improves Cu dispersion or acts as an electronic promoter.^48,49^ Surface Ga_2_O_3_ may simultaneously stabilize Cu^0^ and Cu^I^ species.^19^ This section focuses on the role of Ga, highlighting key similarities and differences with extensively studied Zn promotion.^20–22^

CuGa@SiO_2_, prepared via SOMC and Method A, shows an intrinsic CH_3_OH formation rate (*r*_i‑MeOH_) of 2.8 mol_CH_3_OH_ mol_Cu_^–1^ h^–1^ with ca. 90% CH_3_OH selectivity (Sel_MeOH_), which is substantially higher than the monometallic counterpart, Cu@SiO_2_ (*r*_i‑MeOH_ = 0.8 mol_CH_3_OH_ mol_Cu_^–1^ h^–1^, Sel_MeOH_ = ca. 49%) (Figure 4a and Table S2). *In situ* XAS studies show that under reaction conditions, the CuGa alloy undergoes full dealloying, maximizing the Cu–GaO_*x*_ interfaces and promoting CH_3_OH formation.^1^

**Figure 4:**
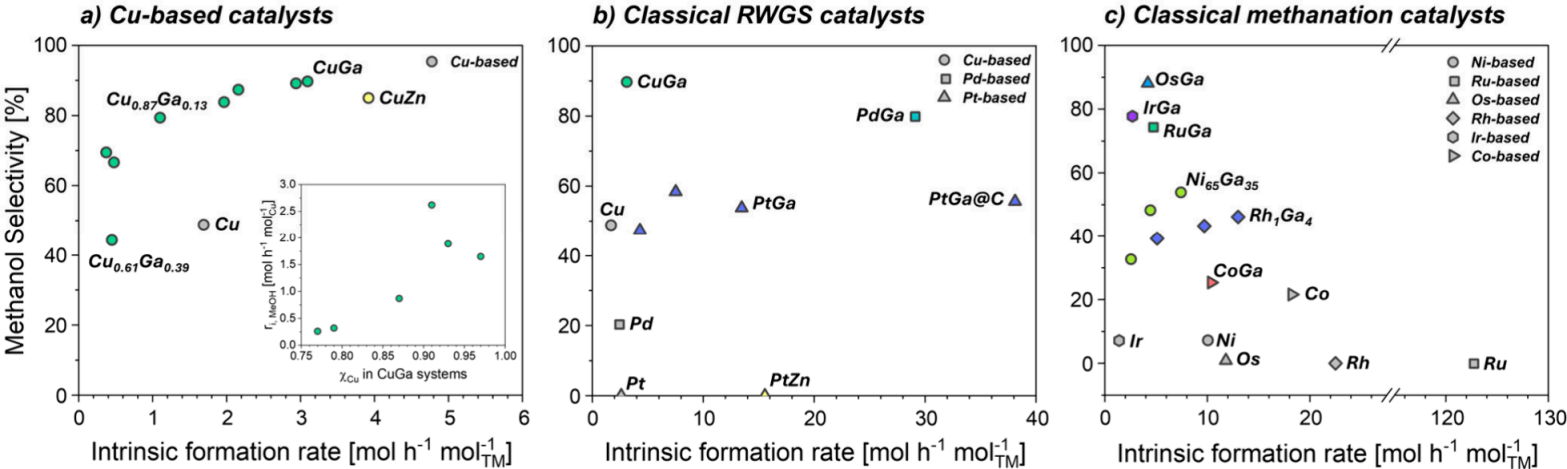
Monometallic vs Ga-doped Cu (a) and Pd (b) and Pt (b) catalysts, primarily RWGS-active when unpromoted; (c) Monometallic vs Ga-doped Ni, Ru, Os, Rh, and Ir catalysts, primarily methanation-active when unpromoted.

Notably the corresponding CuZn@SiO_2_ prepared by the same approach shows slightly higher *r*_i‑MeOH_ = 3.2 mol_CH_3_OH_ mol_Cu_^–1^ h^–1^ and lower Sel_MeOH_ = 86%.^50^ In contrast to the CuGa alloy, the CuZn alloy undergoes only partial dealloying under CO_2_-hydrogenation conditions.

To further elucidate the role of Ga and to minimize the amount of residual Ga^III^ species, a series of Cu_1–*x*_Ga_*x*_@SiO_2_ catalysts with *x* ranging between 0 and 0.39 and particle sizes between 3 and 6 nm have been prepared using a *sequential-grafting* approach (Method B using Ga_2_(O^*t*^Bu)_2_ and Cu_4_(O^*t*^Bu)_4_).^47^ Notably, a volcano-type dependence of the methanol formation rate on Ga content is found for these catalysts. For χ_Ga_ < 0.13–0.18, the material is predicted to form a solid solution according to the Cu–Ga phase diagram (Figure S5). Within this range, the rate increases with increasing Ga content, but it considerably decreases at greater Ga loadings (>18%). Notably, CuGa alloys are predicted according to computations to become less stable at loading above 25% and evolve toward core–shell structures (Figures 4a and S5). Both factors are consistent with the lower catalyst performances of CuGa at higher Ga loadings, highlighting that neither too much nor too little Ga is needed to improve Cu-based catalysts.^51,52^ The “reverse promotional” effect with increasing loading has been linked to the formation of an irreducible GaO_*x*_ shell that encapsulates and poisons the Cu. At low loading, *in situ* XAS shows that GaO_*x*_ in Cu_0.93_Ga_0.07_@SiO_2_ is more redox-active than that in Cu_0.77_Ga_0.23_@SiO_2_ with higher Ga content (Figure 5a). This is due to a lower Ga content, meaning that a higher proportion of Ga participates actively in redox processes, supporting Cu^0^–GaO_*x*_ interface roles. Key methoxy intermediates appear only on Cu_0.93_Ga_0.07_@SiO_2_ (Figure 5b), consistent with its higher activity.

**Figure 5:**
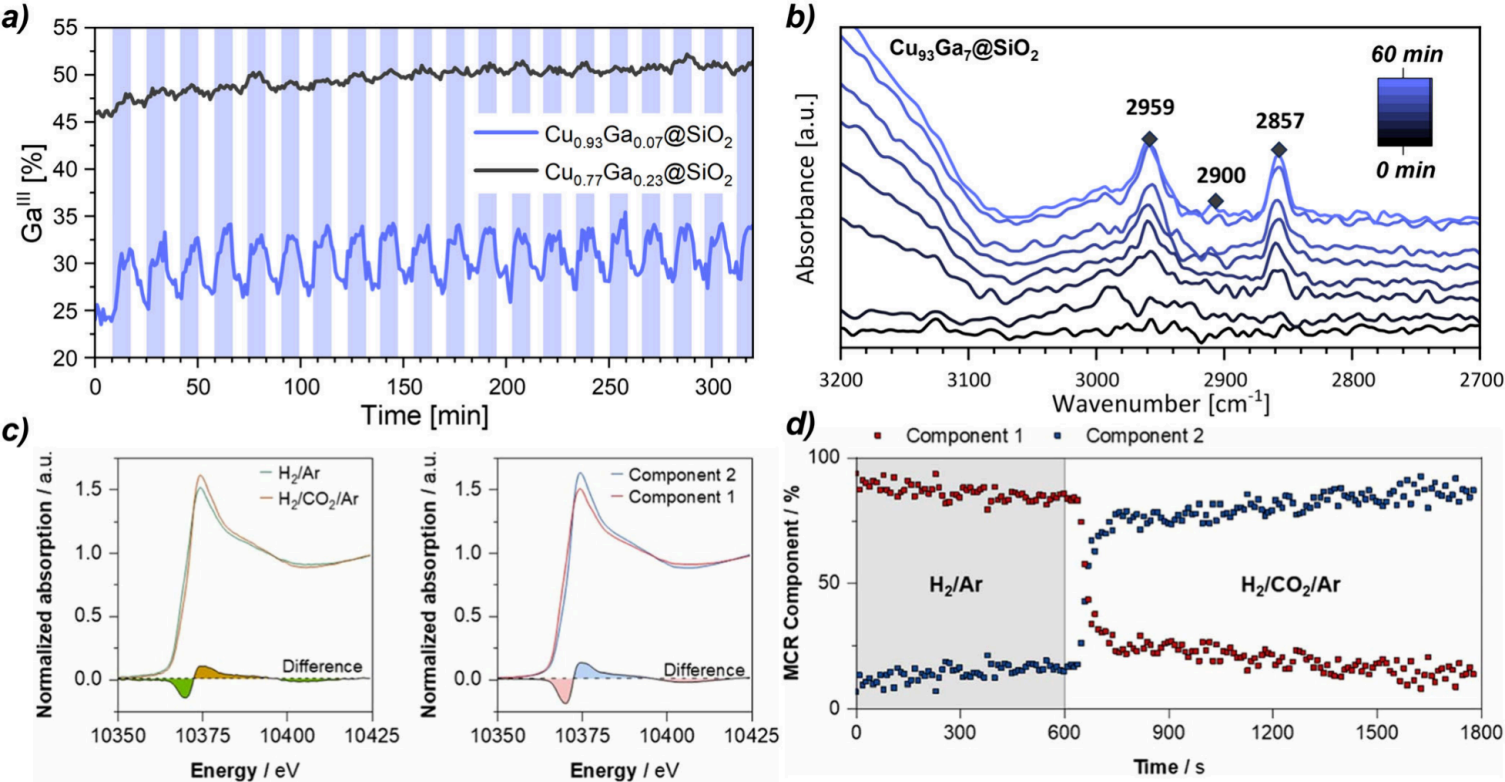
(a) Ga^III^ concentration evolution in Cu_93_Ga_7_@SiO_2_ and Cu_77_Ga_23_@SiO_2_ during gas-switching XANES (230 °C, 5 bar) between H_2_/Ar (3:2) (white) and H_2_/CO_2_/Ar (3:1:1) (blue) (230 °C, 5 bar); (b) Cu_93_Ga_7_@SiO_2_ evolution 1 h after switching from He to H_2_/CO_2_/He (3:1:4) (230 °C, 20 bar) by DRIFTS (adapted from Alfke et al.^47^); (c) Ga K-edge XANES of PdGa@SiO_2_ under reducing/oxidizing conditions (11 bar, 230 °C) with 2-component MCR fit; (d) MCR profiles showing CO_2_ introduction effects over PdGa@SiO_2_ (adapted from Docherty et al.^2^).

DFT calculations and *ab initio* Molecular Dynamics (AIMD) studies reveal that both fcc-CuGa and fcc-CuZn alloys show increased oxygen adsorption compared to pure Cu, which has been attributed to the formation of surface suboxides.^52^ This effect is particularly pronounced in Cu–Ga systems, where surface Ga is predicted to be fully oxidized under both pure CO_2_ and mixed CO_2_/CO hydrogenation conditions. In contrast, the Cu–Zn system shows a more moderate response, with partial dealloying with some Zn retained in the metallic state. This trend aligns with experimental observations where Ga is fully oxidized and Zn is partially oxidized under reaction conditions.

AIMD studies also show that Cu- and Ga-diffusion coefficients in CuGa alloys are higher than those in monometallic systems but lower than those in CuZn.^53^ Ga is a stronger promoter than Zn at low loadings, but its lower mobility can cause Ga-rich surface shells at higher concentrations, leading to oxidation and inhibition of H_2_ activation. Interfaces between GaO_*x*_ and Cu are crucial for CH_3_OH formation but must be tuned to avoid poisoning from less reducible GaO_*x*_ shells at a high Ga loading.

## 4 The Effect of Gallium on Classical RWGS Catalysts: Pd and Pt

While Pd is known to catalyze RWGS, albeit poorly due to CO poisoning, Pd/Ga_2_O_3_ catalysts favor methanol synthesis, surpassing Cu-based systems.^54^ Yet, the specific role of Ga has remained debated in such Pd-based catalysts.^55,56^ Similarly, PdGa@SiO_2_, prepared by Method A and constituted of supported alloyed PdGa nanoparticles, displays a remarkably high intrinsic formation rate and methanol selectivity (*r*_i‑MeOH_ = 22.3 mol_CH_3_OH_ mol_Pd_^–1^ h^–1^, Sel_MeOH_ = 81%; Figure 4a), significantly higher than either CuGa@SiO_2_ (2.8 mol_CH_3_OH_ mol_Cu_^–1^ h^–1^, Sel_MeOH_ = ca. 90%) or Pd@SiO_2_ (0.51 mol_CH_3_OH_ mol_Pd_^–1^ h^–1^, Sel_MeOH_ = 20%). *In situ* XAS at the Ga K-edge reveals that part of Ga is dealloyed and oxidized to form highly dispersed Ga^III^O_*x*_ under reaction conditions. Notably, gas-switching XAS and DRIFTS experiments suggest that an increased proportion of Ga^0^ is observed under H_2_-rich conditions, with a concomitant emergence of formate species.^2^ This study indicates that formate surface species are preferentially formed when the catalyst is under a more reduced state, while under CO_2_-rich, oxidizing conditions, an increased proportion of tetracoordinate Ga^III^O_*x*_ is observed, alongside an increased amount of surface methoxy species.

These findings suggest that there is a dynamic evolution of PdGa alloy nanoparticles and that the predominant surface intermediates depend on the state of the catalyst, which is itself driven by the ratio of H_2_/CO_2_. AIMD/MTD simulations show that PdGa alloying creates isolated Pd sites, altering CO and H adsorption versus pure Pd. Adsorbed H is more hydridic on PdGa, suggesting higher CO_2_ reactivity.^57^ Under oxidizing conditions, the alloy forms a Pd-rich core with partially oxidized GaO_*x*_ surface sites, paralleling Cu systems where metal–GaO_*x*_ interfaces and redox processes enhance methanol selectivity.

Regarding Pt-based catalysts, that are ubiquitous for low temperature RWGS, only a few examples have been discussed in the context of methanol synthesis, focusing on Cr- or W-promotion.^58^ Notably, PtGa@SiO_2_, also prepared by Method A, shows a methanol formation rate of 7.2 mol_CH_3_OH_ mol_Pt_^–1^ h^–1^ (Figure 4b) with Sel_MeOH_ close to 50%, sharply contrasting with Pt@SiO_2_, associated with poor RWGS activity and no methanol formation. *In situ* XAS and DRIFTS studies indicate that PtGa catalysts slightly dealloy under CO_2_ hydrogenation conditions to generate PtGa–GaO_*x*_ interfaces. Their formation correlates with increased CO_2_-hydrogenation activity and methanol selectivity. Similarly, the presence of Ga enables the observation of formate and methoxy species, both key reaction intermediates, which are overall connected to increase methanol formation rates and selectivities. It is noteworthy that the corresponding PtZn@SiO_2_ shows almost exclusive CO selectivity (∼99%), sharply contrasting with Ga-based systems. *In situ* spectroscopic studies complemented with DFT calculations reveal that the surface of the PtZn alloy remains stable without undergoing surface oxidation under CO_2_ hydrogenation, resulting in the decomposition of formate (HCOO*) species to produce CO.^59^ Therefore, these findings strongly suggest that the interface between alloy nanoparticles and GaO_*x*_ is crucial in promoting CH_3_OH synthesis.

Noteworthily, the corresponding carbon supported PtGa catalysts, prepared via SOMC using *sequential grafting* (Method B), show an increased methanol formation rate (21.2 mol_CH_3_OH_ mol_Pt_^–1^ h^–1^) with a similar CH_3_OH selectivity compared to PtGa@SiO_2_ (Figure 4b). This result indicates that the absence of Lewis acidic Ga^III^-sites, not formed on carbon, might facilitate CH_3_OH desorption and overall enhanced methanol formation rates,^3^ possibly indicating that increasing redox dynamics between MGa and GaO_*x*_ could be important to drive overall reaction rates.

## 5 The Effect of Gallium on Classical Methanation Catalysts

Other transition metals, Fe, Co, Ni, Ru, Rh, Os, and Ir, are known for methanation. Silica-supported nanoparticles prepared via SOMC produce CH_4_ with approximately 100% selectivity under CO_2_-hydrogenation (Figure 4b). In contrast, Ni–Ga alloys, stable under reaction conditions, yield methanol, highlighting the positive effect of alloying.^60^ A series of silica-supported NiGa catalysts with various Ni/Ga ratios have thus been prepared via SOMC (Method A) and evaluated in CO_2_ hydrogenation. The most selective system is Ni_65_Ga_35_@SiO_2_ that displays a methanol formation rate of ca. 4.0 mol_MeOH_ mol_Ni_^–1^ h^–1^ and Sel_MeOH_ = 53.9%, with no methane formation (Sel < 1%). This sharply contrasts with the high methane selectivity (ca. 88%) and CO_2_ conversion rate (10.1 mol_CO_2__ mol_Ni_^–1^ h^–1^) of the monometallic catalyst (Figure 4c). Note that other compositions display catalytic activity and selectivity significantly below that of Ni_65_Ga_35_@SiO_2_.^42^ Detailed spectroscopic investigations using *in situ* differential pair distribution function analysis (d-PDF) and XAS after catalyst preactivation in hydrogen at 300 °C reveal the formation of a Ni_3_Ga alloy with a random fcc arrangement, owing to the small nanoparticle size of approximately 2 nm.

However, while *in situ* XAS and EXAFS analyses reveal that the bulk of the Ni_3_Ga alloy is stable throughout CO_2_-hydrogenation, a fraction of gallium, most likely at the surface, is partially oxidized. Conversely, surface sensitive *in situ* DRIFTS experiments highlight the formation of adsorbed CO* and methoxy (−OCH_3_*) species associated with an intense peak at 2054 cm^–1^ and two minor peaks at 2962 and 2860 cm^–1^, respectively (Figure 6a,b), which is strongly related to the mechanisms over Cu, Pd, and Pt.

**Figure 6:**
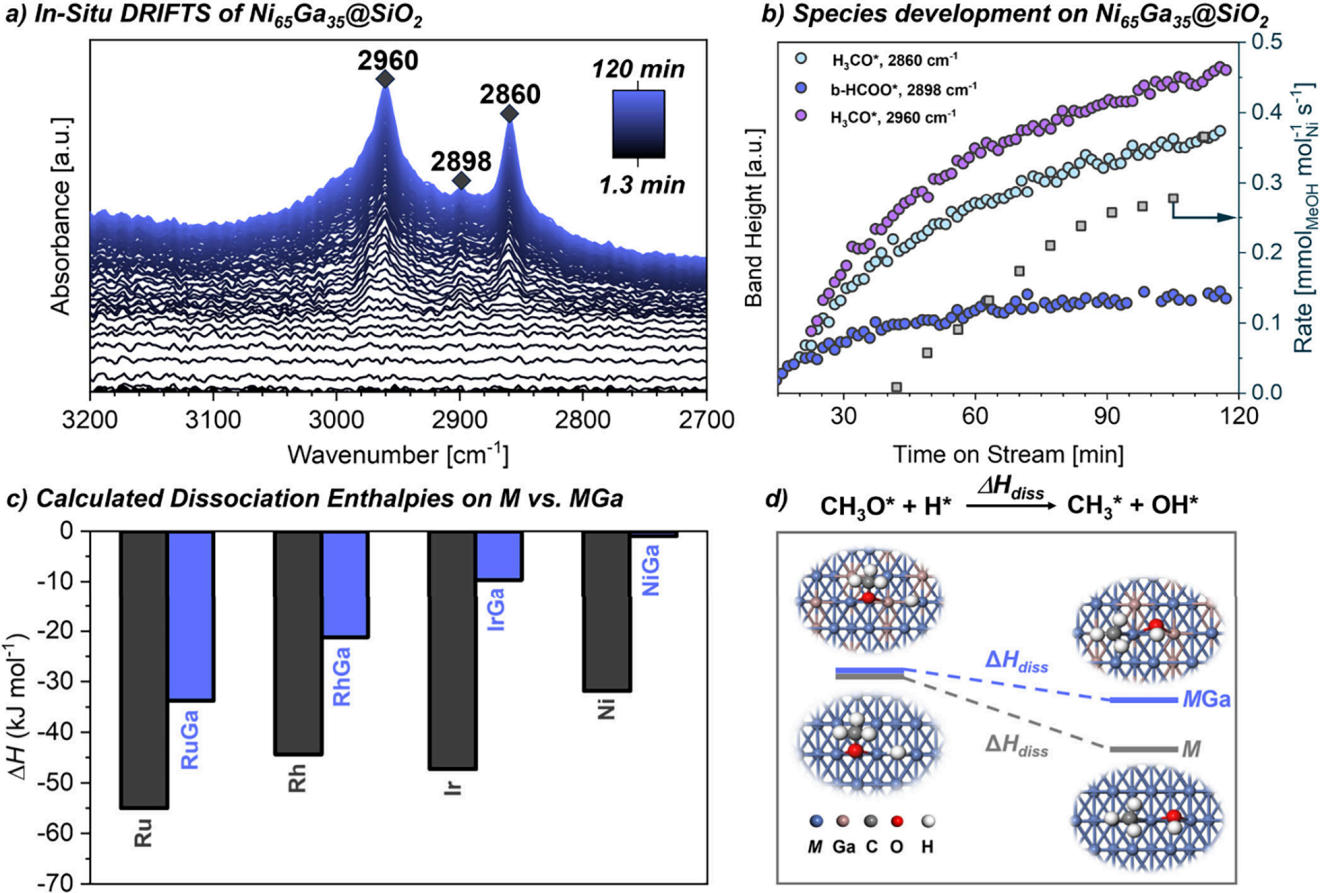
(a) *Operando* DRIFTS of Ni_65_Ga_35_@SiO_2_ (20 bar, CO_2_/H_2_/N_2_ = 1:3:1, 230 °C, GHSV = 60 L g_cat_^–1^ h^–1^) (time scale denotes time after switching from pure N_2_ to reaction conditions); (b) Species development (from DRIFTS) and methanol formation rate (adapted from Zimmerli et al.^42^); (c) Calculated C–O dissociation energies in methoxy species adsorbed on the surface of mono- and bimetallic catalysts; (d) Visual representation of the M–C–O dissociation process (adapted from Zhou et al.^4^).

However, the silica-supported FeGa and CoGa systems show low methanol formation rates and selectivity, likely because of the propensity for these elements to readily oxidize^39^ under CO_2_ hydrogenation conditions, correlating with their RWGS activity (Figure 4b).

For noble elements (M = Ru, Os, Rh, or Ir, prepared by method A), all MGa@SiO_2_ show high methanol selectivities of ca. 74%, 88%, 39%, and 78%) with total intrinsic formation rates of 4.7, 4.2, 5.1, and 2.7 mol_CO_2__ mol_TM_^–1^ h^–1^. These results sharply contrast with the monometallic systems that show nearly 100% CH_4_ selectivity and high activity up to 122.0 mol_CH_4__ mol_Ru_^–1^ h^–1^ for Ru (Figure 4c and Table S2). HAADF-STEM, EDX, and XAS (including EXAFS fitting) evidence the formation of a M–Ga alloy in all cases, with M/Ga ratios of 1.6:1–3.0:1 as determined by LCF-analysis (further evidenced by a negative energy of formation for all the alloys, as calculated by DFT).^4^ *In situ* XAS reveals that the alloys that formed upon catalyst preactivation in H_2_ at 400 °C are stable throughout reaction conditions. Additionally, the same features as in the NiGa systems are observed via *in situ* DRIFTS experiments: one can identify M–CO* (2037–2063 cm^–1^), as well as peaks at ca. 2960 cm^–1^ and 2860^–1^, indicating that the M–Ga alloys of Ni, Ru, Rh, Os, and Ir behave similarly.

The origin of Ga-promotion for the methanol synthesis using late-transition metals is interrogated via DFT calculations. Highly negative dissociation enthalpies (Δ*H*_diss_) suggest easier C–O bond cleavage on pure metals, while the addition of Ga significantly decreases these negative values thereby increasing the stability of methoxy species. The results show that the Δ*H*_diss_ on monometallic surfaces is generally more favorable than on a M–Ga surface (Figure 6c,d)^4^ and imply that the incorporation of Ga stabilizes methoxy species, thereby promoting methanol selectivity.

Overall, the suppression of methanation activity and the concomitant methanol formation are most likely due to (i) the formation of stable alloys that prevent large metallic ensembles active in methanation and (ii) the formation of GaO_*x*_–MGa interfaces at the surface of the particles, thus facilitating the formation of methoxy species, as observed and proposed for the other systems (Cu, Ni, Pd, and Pt).

## 6 Conclusions and Perspective

This Account highlights the universal role of Ga in promoting methanol synthesis in CO_2_-hydrogenation across late transition metals. The systems have been investigated by advanced *in situ* and *operando* characterization techniques giving insights to structural dynamics. M–Ga alloys, formed under H_2_ treatment, partially or fully dealloy under reaction conditions (230 °C, 20–40 bar, 1:3 CO_2_/H_2_), the extent of which depends on the M: CuGa fully dealloys, PdGa partially dealloys, and PtGa only slightly dealloys. The other metals known for their methanation activity (Ni, Ru, Os, Rh, Ir) retain stable bulk MGa alloys, essential for suppressing methanation, while a small fraction of MGa–GaO_*x*_ also turns on methanol selectivity. Note that Fe and Co fully dealloy and oxidize under these conditions, hence explaining the low methanol selectivity. Overall, MGa–GaO_*x*_ or M–GaO_*x*_ interfacial sites are crucial for methanol synthesis (Figure 7). However, this universal increase in methanol selectivity has different origins (Figure S1): for methanation catalysts, it is linked to stable bulk alloy formation, which reduces both methane formation and the CO_2_ conversion rate for most systems (Ru, Os, Rh, Ni), Ir being an exception, where Ga also increases the activity. This shows that Ga poisons methanation sites upon alloying, while MGa−GaO_*x*_ promotes methanol formation. For Cu or RWGS elements (Pd, Pt), dealloying generates M–GaO_*x*_ interfaces that increase both CO_2_ conversion rate and methanol selectivity compared to pure metals, indicating a “true” promotional effect. Calculations suggest that the presence of Ga increases the hydricity of M­(Pd)–H, possibly explaining the increased activity. These interfaces are dynamic under reaction conditions, according to gas-switching experiments (CO_2_ vs CO_2_/H_2_).

**Figure 7:**
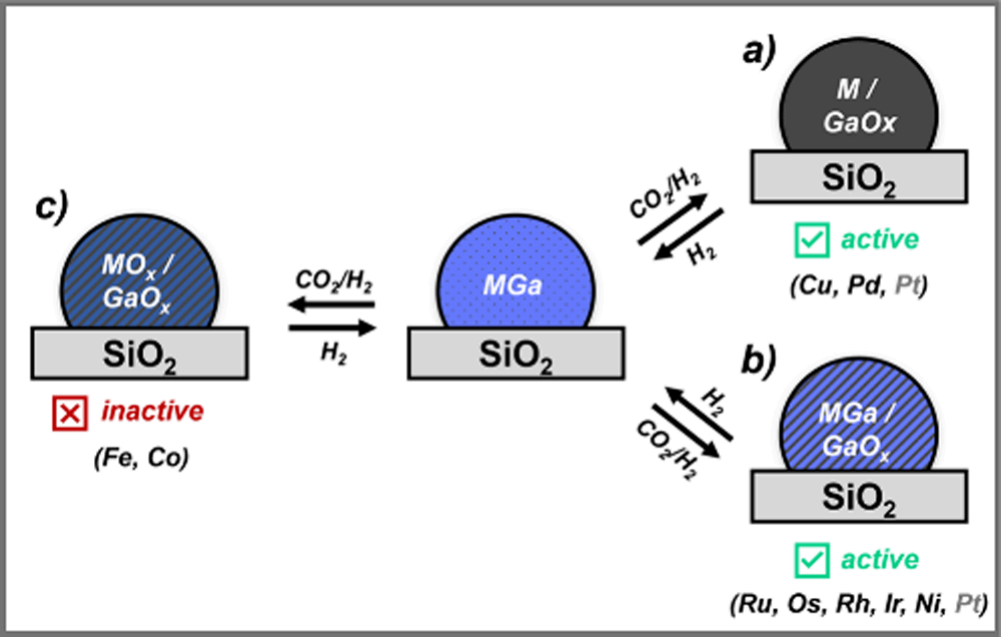
Extent of dealloying depends on the nature of M, and the formation of GaO_*x*_ is key to promoting methanol synthesis: (a) Cu and metals known for RGWS (Pd, Pt) generate M–GaO_*x*_ interfaces; (b) Metals known for methanation (Ni, Ru, Os, Rh, Ir) generate stable bulk alloys with Ga and MGa/GaO_*x*_ interfaces, preventing methanation; (c) Fe and Co are readily oxidized and are not promoted by Ga.

While this Account focused on CO_2_ hydrogenation, future studies should examine a greater range of conditions, closely related to what is being used in industry (e.g., CO/CO_2_ mixtures), to further elaborate the structure–reactivity relationships and identify in greater detail the role of the gas phase composition on the state and the reactivity of the active phase. While all studied materials show no major deactivation, long-term stability tests and understanding related deactivation processes would be necessary in future studies. Notably, while MGa–GaO_*x*_ and M–GaO_*x*_ dynamics are probably important, the connection of the observed reactivity to dynamic phenomena remains to be understood. This will require more detailed *in situ* and *operando* techniques (ambient-pressure XPS, *in situ* STEM, gas-switching XAS) and kinetic studies complemented by computational investigations. Such insights will help identify the role of active site structure and dynamics and possibly identify selectivity gains by non-steady-state regimes.^61,62^ It is likely that the observed dynamics can also be affected by the support across MGa systems, as already illustrated for PtGa on silica and carbon (vide supra)^3^ or earlier works, where support effects are observed for Cu nanoparticles alone,^41,63^ clearly pointing out to the need to expand this study to understand the role of the support in driving the overall catalytic performance of these complex systems.

Similar to the aforementioned Ga- and Zn-promoted systems, intermetallic In systems have also shown favorable properties for methanol synthesis. Considering the similarities between Ga, Zn, and In, all forming reducible oxides that can activate H_2_ and CO_2_,^64–66^ in-depth studies of MIn systems would be important steps to expand our knowledge on the role of reducible promoters.

A final point is the universality of Ga-promotion across elements compared to Zn; this seems to parallel the greater stability of Zn alloys, which will affect the types of interfaces under reaction conditions. All in all, bi- and multimetallic systems remain poorly understood, and data-driven high throughput experiments (HTE) involving synthesis, testing, characterization, and computations are emerging as a powerful tool for the systematic investigation of the chemical space.

## Supplementary Material



## Notes

Additional catalyst properties, performances and metrics, as well as thermodynamic analyses and phase diagrams (PDF)
