# Supplementary material for: The Universal Role of Gallium in Promoting Methanol Formation across CO2 Hydrogenation Catalysts
Source: Acc Chem Res. 2025 Nov 6;58(22):3392–401. doi: 10.1021/acs.accounts.5c00581 (PMC12631983; doi:10.1021/acs.accounts.5c00581)
Supplement: Supplementary file 1 [file ar5c00581_si_001.pdf]

SUPPORTING INFORMATION

The Universal Role of Gallium in Promoting Methanol Formation Across CO_2 Hydrogenation Catalysts

Colin Hansen[a], Wei Zhou[a,*] and Christophe Copéret[a,*]

[a] Department of Chemistry and Applied Biosciences, ETH Zürich, Vladimir Prelog Weg 1-5, CH-8093 Zurich, Switzerland

*Corresponding Authors: weizhou@ethz.ch; ccoperet@ethz.ch

Contents

S1 Supplementary Data **2**

References **10**

S1 Supplementary Data

Table S 1. Summary of physicochemical properties of mono- and bimetallic catalysts with different M/Ga ratios prepared by a single-site approach (Method A). Italic values are estimated by the synthetic procedure (nominal loading).

Catalyst	Metal loading (wt%)[a]	M/Ga ratio[a]	Particle size (nm)[b]	Ga proportion (%)	M/Ga^0 ratio in alloy[c]
Cu@SiO_2	Cu: 4.6	-	2.9 ± 1.3	-	-
CuGa@SiO_2	Cu: 3.88<br>Ga: 1.61	2.64:1	4.6 ± 1.4	Ga^{III}: 50<br>Ga^0: 50	5:1
CuZn@SiO_2	Cu: 4.16<br>Zn: 1.62	2.64:1	3.9 ± 1.0	Zn^{II}: 71<br>Zn^0: 29	9.1:1
Pd@SiO_2	Pd: 2.9	-	1.6 ± 0.3	-	-
PdGa@SiO_2	Pd: 1.08<br>Ga: 1.66	0.40:1	1.6 ± 0.4	Ga^{III}: 33<br>Ga^0: 77	0.6:1
Pt@SiO_2	Pt: 4.18	-	1.9 ± 0.6	-	-
PtGa@SiO_2	Pt: 4.53<br>Ga: 1.70	0.95:1	1.9 ± 0.8	Ga^{III}: 47<br>Ga^0: 53	1.8:1
Pt_1Ga_2@SiO_2	Pt: 2.64<br>Ga: 1.77	0.53:1	1.8 ± 0.4	Ga^{III}: 49<br>Ga^0: 51	1.0:1
Pt_1Ga_4@SiO_2	Pt: 1.30<br>Ga: 1.77	0.26:1	1.7 ± 0.4	Ga^{III}: 44<br>Ga^0: 56	0.5:1
PtZn@SiO_2	Pt: 4.40<br>Zn: 1.75	0.84:1	1.8 ± 0.7	Zn^{II}: 20<br>Zn^0: 80	1.0:1
Ni@SiO_2	Ni: 2.12	-	2.0 ± 0.7	-	-
$Ni_{75}Ga_{25}$@SiO_2	Ni: 2.08<br>Ga: 0.76	3.17:1	2.4 ± 0.7	Ga^{III}: 29<br>Ga^0: 71	4.5:1
$Ni_{70}Ga_{30}$@SiO_2	Ni: 2.21<br>Ga: 1.03	2.57:1	2.6 ± 0.8	Ga^{III}: 12<br>Ga^0: 88	3.0:1
$Ni_{65}Ga_{35}$@SiO_2	Ni: 2.17<br>Ga: 1.24	2.03:1	2.3 ± 0.7	Ga^{III}: 29<br>Ga^0: 71	2.8:1
Ru@SiO_2	Ru: 2.26	-	2.0 ± 0.9	-	-
RuGa@SiO_2	Ru: 2.84<br>Ga: 1.74	1.13:1	1.7 ± 0.7	Ga^{III}: 63<br>Ga^0: 37	3.0:1
Os@SiO_2	Os: 3.23	-	1.6 ± 0.6	-	-

OsGa@SiO_2	Os: 4.56<br>Ga: 1.54	1.07:1	1.8 ± 0.7	-	-
Fe@SiO_2	Fe: *1.5*	-	1.5 ± 0.2	-	-
FeGa@SiO_2	Fe: *1.5*<br>Ga: *1.6*		4.2 ± 1.1	-	-
Rh@SiO_2	Rh: 3.23	-	3.0 ± 1.0	-	-
RhGa@SiO_2	Rh: 2.50<br>Ga: 1.78	0.95:1	1.7 ± 0.7	Ga^{III}: 39<br>Ga^{0}: 61	1.6:1
Rh_1Ga_2@SiO_2	Rh: 1.16<br>Ga: 1.87	0.42:1	1.3 ± 0.6	-	-
Rh_1Ga_4@SiO_2	Rh: 0.52<br>Ga: 1.89	0.18:1	0.8 ± 0.3	-	-
Ir@SiO_2	Ir: 4.89	-	2.1 ± 0.8	-	-
IrGa@SiO_2	Ir: 4.63<br>Ga: 1.56	1.09:1	1.6 ± 0.6	Ga^{III}: 55<br>Ga^{0}: 45	2.4:1
Co@SiO_2	Co:1.64	-	3.0 ± 1.0	-	-
CoGa@SiO_2	Co: 1.6<br>Ga: 1.7	-	1.8 ± 0.4	-	-

[a] Determined by element analysis (EA); [b] Particle size determined by STEM.

Table S 2. Summary of physicochemical properties of mono- and bimetallic catalysts with different M/Ga ratios prepared by a sequential grafting approach (Method B). Italic values are estimated based on coordination number from EXAFS fitting.

**Catalyst**	**Metal loading (wt%)**[a]	**M/Ga ratio**[a]	**Particle size (nm)**[b]	**Ga proportion (%)**	**M/Ga^0 ratio in alloy**[c]
$Cu@SiO_2$	Cu: 4.6	-	2.9 ± 1.3	-	-
$Cu_{0.97}Ga_{0.03}@SiO_2$	Cu: 2.6<br>Ga: 0.1	28.6:1	3	Ga^{III}: 18<br>Ga^0: 82	34.9:1
$Cu_{0.93}Ga_{0.07}@SiO_2$	Cu: 3.7<br>Ga: 0.3	13.5:1	3.5	*Ga^{III}: 18*<br>*Ga^0: 82*	16.4:1
$Cu_{0.91}Ga_{0.09}@SiO_2$	Cu: 3.6<br>Ga: 0.4	9.86:1	4.8	*Ga^{III}: 18*<br>*Ga^0: 82*	12:1
$Cu_{0.87}Ga_{0.13}@SiO_2$	Cu: 3.1<br>Ga: 0.5	6.80:1	4.4	-	-
$Cu_{0.79}Ga_{0.21}@SiO_2$	Cu: 4.1<br>Ga: 1.2	3.75:1	4.2	*Ga^{III}: 20*<br>*Ga^0: 80*	4.6:1
$Cu_{0.77}Ga_{0.23}@SiO_2$	Cu: 2.5<br>Ga: 0.8	3.43:1	6.5	Ga^{III}: 50<br>Ga^0: 50	6.9:1
$Cu_{0.61}Ga_{0.39}@SiO_2$	Cu: 2.7<br>Ga: 1.9	1.56:1	5.5	Ga^{III}: 23<br>Ga^0: 77	2.0:1
$Pt@SiO_2$	Pt: 4.18	-	1.9 ± 0.6	-	-
PtGa@C	Pt: 0.26<br>Ga: 0.10	0.93:1	1.4 ± 0.5	-	0.9:1

[a] Determined by element analysis (EA); [b] Particle size determined by STEM.

Table S 3. Summary of intrinsic formation rate and selectivities of different catalysts for CO_2 hydrogenation.

Catalyst	Intrinsic formation rate ($mol\ h^{-1}\ mol_{TM}^{-1}$)				Intrinsic CH_4 Select.[d] (%)	Intrinsic CO Select.[d] (%)	Intrinsic CH_3OH Select.[d] (%)
	CH_4	CO	CH_3OH	Total[d]			
Cu@SiO_2[a]	0	0.86	0.83	1.69 / *0.83*	0 / *0*	51.1	48.9 / *100*
CuGa@SiO_2[a]	0	0.32	2.78	3.10 / *2.78*	0 / *0*	10.3	89.7 / *100*
$Cu_{0.97}Ga_{0.03}$-SiO_2[a]	0	0.32	1.65	1.97 / *1.65*	0 / *0*	16.2	83.8 / *100*
$Cu_{0.93}Ga_{0.07}$-SiO_2[a]	0	0.27	1.89	2.16 / *1.89*	0 / *0*	12.6	87.4 / *100*
$Cu_{0.91}Ga_{0.09}$-SiO_2[a]	0	0.32	2.62	2.94 / *2.62*	0 / *0*	10.8	89.2 / *100*
$Cu_{0.87}Ga_{0.13}$-SiO_2[a]	0	0.23	0.87	1.10 / *0.87*	0 / *0*	20.6	79.4 / *100*
$Cu_{0.77}Ga_{0.23}$-SiO_2[a]	0	0.11	0.26	0.37 / *0.26*	0 / *0*	30.5	69.5 / *100*
$Cu_{0.79}Ga_{0.21}$-SiO_2[a]	0	0.16	0.32	0.48 / *0.32*	0 / *0*	33.3	66.7 / *100*
$Cu_{0.61}Ga_{0.39}$-SiO_2[a]	0	0.25	0.20	0.45 / *0.20*	0 / *0*	55.7	44.3 / *100*
CuZn@SiO_2[a]	0	0.59	3.33	3.92 / *3.33*	0 / *0*	15.0	85.0 / *100*
Pd@SiO_2[a]	0.11	1.80	0.50	2.41 / *0.61*	4.6 / *18.2*	74.7	20.7 / *81.8*
PdGa@SiO_2[a]	0.10	5.70	23.20	29.00 / *23.30*	0.3 / *0.4*	19.7	80.0 / *99.6*
Pt@SiO_2[a]	0.87	1.73	0	2.60 / *0.87*	33.5 / *100*	66.5	0 / *0*
PtGa@SiO_2(1:1)[a]	0.09	6.14	7.24	13.5 / *7.33*	0.7 / *1.3*	45.6	53.7 / *98.7*
PtGa@SiO_2(0.5:1)[a]	0.15	2.98	4.39	7.52 / *4.54*	2.0 / *3.3*	39.6	58.4 / *96.7*
PtGa@SiO_2(0.25:1)[a]	0.05	2.21	2.03	4.29 / *2.08*	1.2 / *2.5*	51.5	47.3 / *97.5*
PtZn@SiO_2[a]	0.06	15.5	0	15.56 / *0.06*	0.4 / *100*	99.6	0 / *0*
PtGa@C[a]	0.51	16.4	21.2	38.11 / *21.71*	1.4 / *2.5*	43.0	55.6 / *97.5*
Ni@SiO_2[b]	8.80	0.52	0.72	10.05 / *9.52*	87.6 / *92.4*	5.2	7.2 / *7.6*
$Ni_{75}Ga_{25}$@SiO_2 [b]	0.28	1.44	0.84	2.55 / *1.12*	11.1 / *25.3*	56.2	32.7 / *74.7*
$Ni_{70}Ga_{30}$@SiO_2 [b]	0.01	2.28	2.13	4.44 / *2.14*	0.3 / *0.6*	51.5	48.2 / *99.4*

$Ni_{65}Ga_{35}$@SiO_2 [b]	0	3.42	4.00	7.42 / *4.00*	0 / *0*	46.1	53.9 / *100*
Ru@SiO_2[c]	121.8	0.4	0.4	122.6 / *122.2*	99.4 / *99.7*	0.3	0.3 / *0.3*
RuGa@SiO_2[c]	0.3	0.9	3.5	4.7 / *3.8*	6.4 / *7.9*	19.1	74.5 / *92.1*
Os@SiO_2[c]	11.6	0.1	0.1	11.8 / *11.7*	98.4 / *99.2*	0.8	0.8 / *0.8*
OsGa@SiO_2[c]	0.1	0.4	3.7	4.2 / *3.8*	2.4 / *2.7*	9.5	88.1 / *97.3*
Fe@SiO_2[c]	0.3	0.6	0.1	1 / *0.4*	30.0 / *75.0*	60.0	10.0 / *25.0*
FeGa@SiO_2[c]	0.1	0.8	0.1	1 / *0.2*	10.0 / *50.0*	80.0	10.0 / *50.0*
Rh@SiO_2[c]	22.1	0.4	0	22.5 / *22.1*	98.2 / *100*	1.8	0 / *0*
RhGa@SiO_2[c]	0.1	3.0	2.0	5.1 / *2.1*	2.0 / *4.9*	58.8	39.2 / *95.1*
Rh_1Ga_2@SiO_2[c]	0.2	5.3	4.2	9.7 / *4.4*	2.1 / *4.6*	54.6	43.3 / *95.4*
Rh_1Ga_4@SiO_2[c]	0.2	6.8	6.0	13.0 / *6.2*	1.5 / *3.1*	52.3	46.2 / *96.9*
Ir@SiO_2[c]	1.2	0.1	0.1	1.4 / *1.3*	85.8 / *92.4*	7.1	7.1 / *7.6*
IrGa@SiO_2[c]	0	0.6	2.1	2.7 / *2.1*	0 / *0*	22.2	77.8 / *100*
Co@SiO_2[a]	9.1	5.2	3.9	18.2 / *13.0*	50.1 / *69.9*	28.3	21.6 / *30.1*
CoGa@SiO_2[a]	1.6	6.2	2.6	10.4 / *4.2*	14.9 / *27.0*	59.7	25.4 / *63.0*

[a] Reaction conditions: F = 6-100 mL/min, T = 230 ºC, P = 25 bar.

[b] The formation rate and selectivity was obtained at 60000 ml $h^{-1}g_{cat}^{-1}$. F = 100 mL/min, T = 230 ºC, P = 25 bar.

[c] Reaction conditions: F = 6-100 mL/min, T = 230 ºC, P = 40 bar.

[d] Formation rates and selectivities excluding CO as a product given in italic.

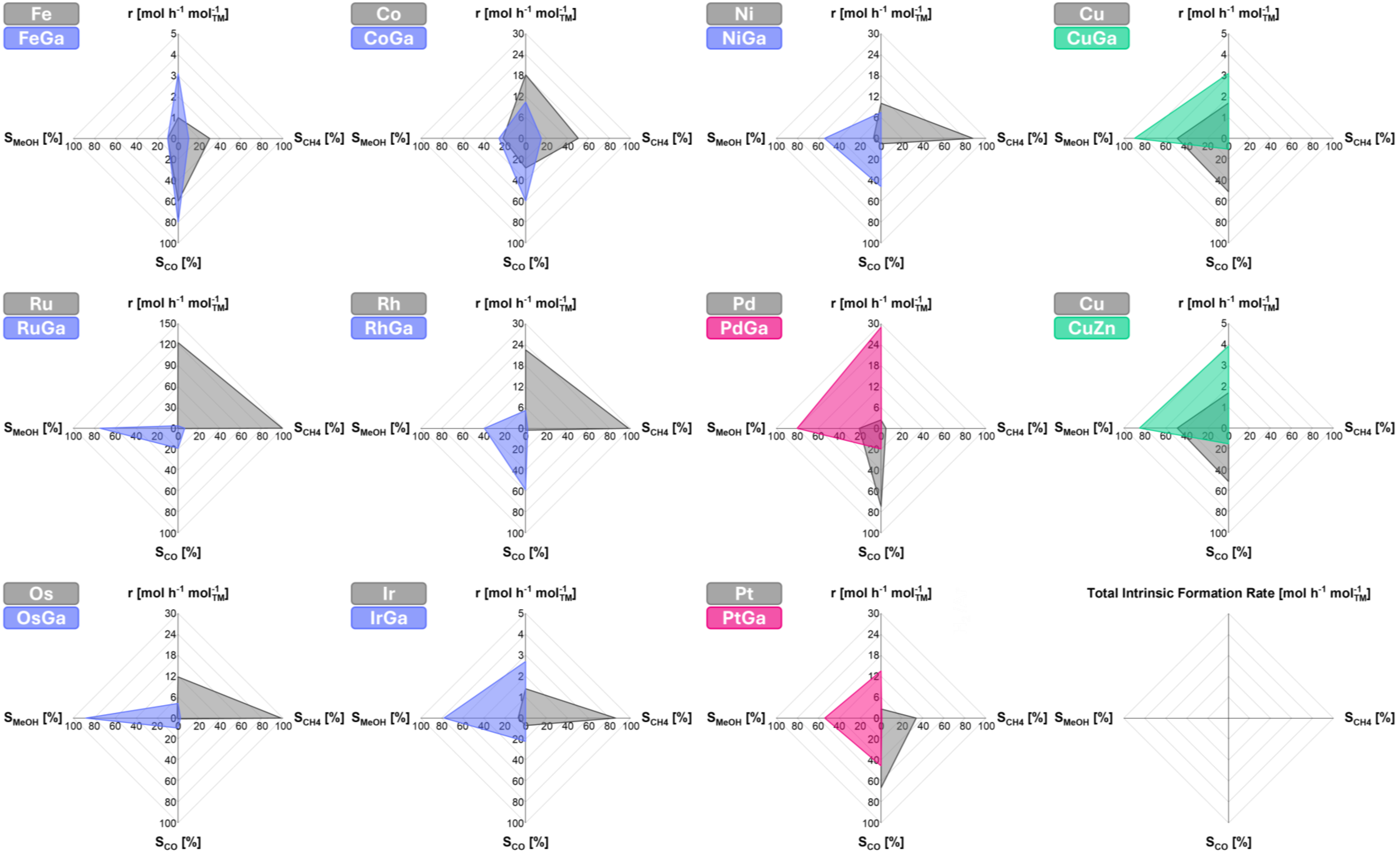

Figure S 1. Radar plots for mono- and bimetallic systems showing the unique promotional effect of gallium. $Ni_{65}Ga_{35}$ was chosen as the representative catalyst for NiGa. (r = total intrinsic formation rate). Data from Table S1.

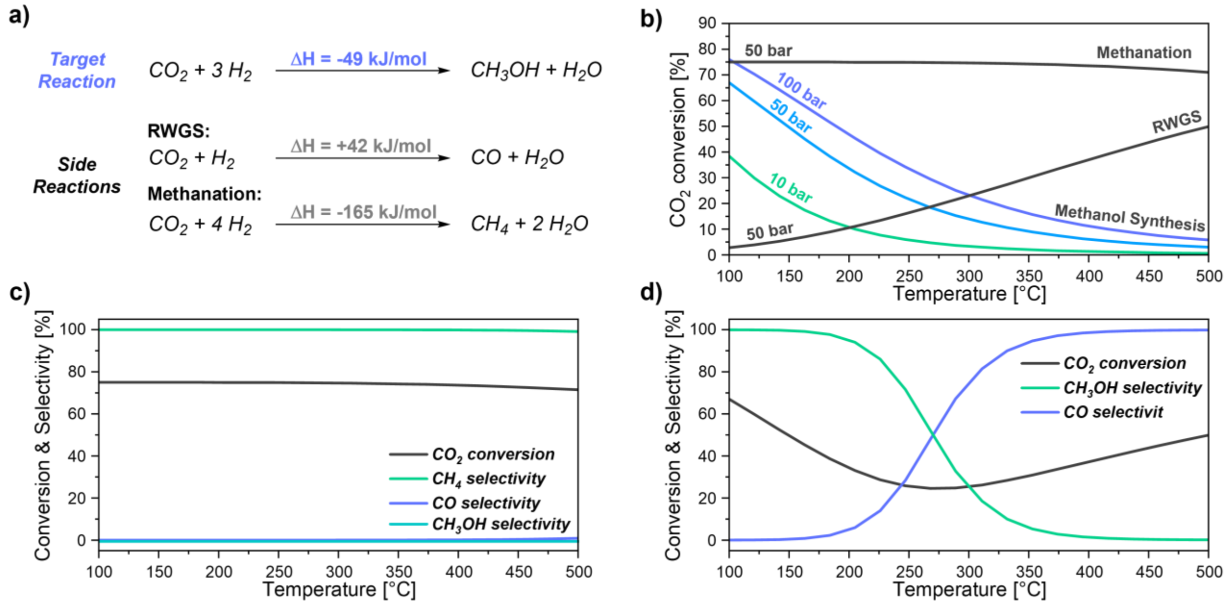

Figure S 2. a) Reaction enthalpies of the most common reactions in CO_2 Hydrogenation; b) Thermodynamic CO_2 conversion for the three main reactions in a 1:3:1 CO_2/H_2/Ar mix; c) Selectivities and conversion vs. temperature at 50 bars; d) Selectivities and conversion vs. temperature at 50 bars (excluding methanation).

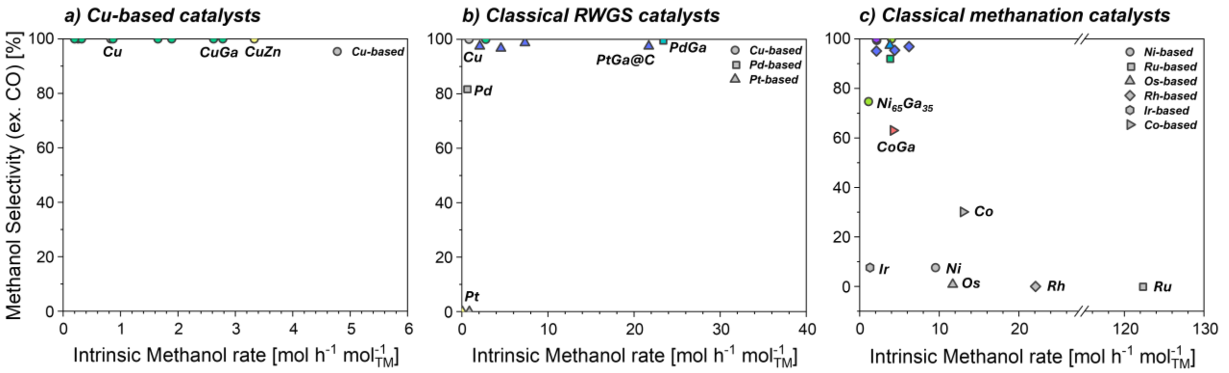

Figure S 3. Selectivity vs. Methanol formation rate (excluding CO as a product) for all the systems listed in Table S2. (a) Cu-based systems (Cu = grey circle, CuGa = green circles, CuZn = yellow circle); (b) RWGS systems (Cu = grey circle, CuGa = green circle, Pd = square, PdGa = cyan square, Pt = grey triangle, PtGa = blue triangles, PtZn = yellow triangle); (c) Methanation systems (Ni = grey circle, NiGa = green circle, Ru = grey square, RuGa = turquoise square, Os = grey triangle, OsGa = cyan triangle, Rh = grey rhomboid, RhGa = blue rhomboids, Ir = grey hexagon, IrGa = purple hexagon, Co = grey arrow, CoGa = red arrow).

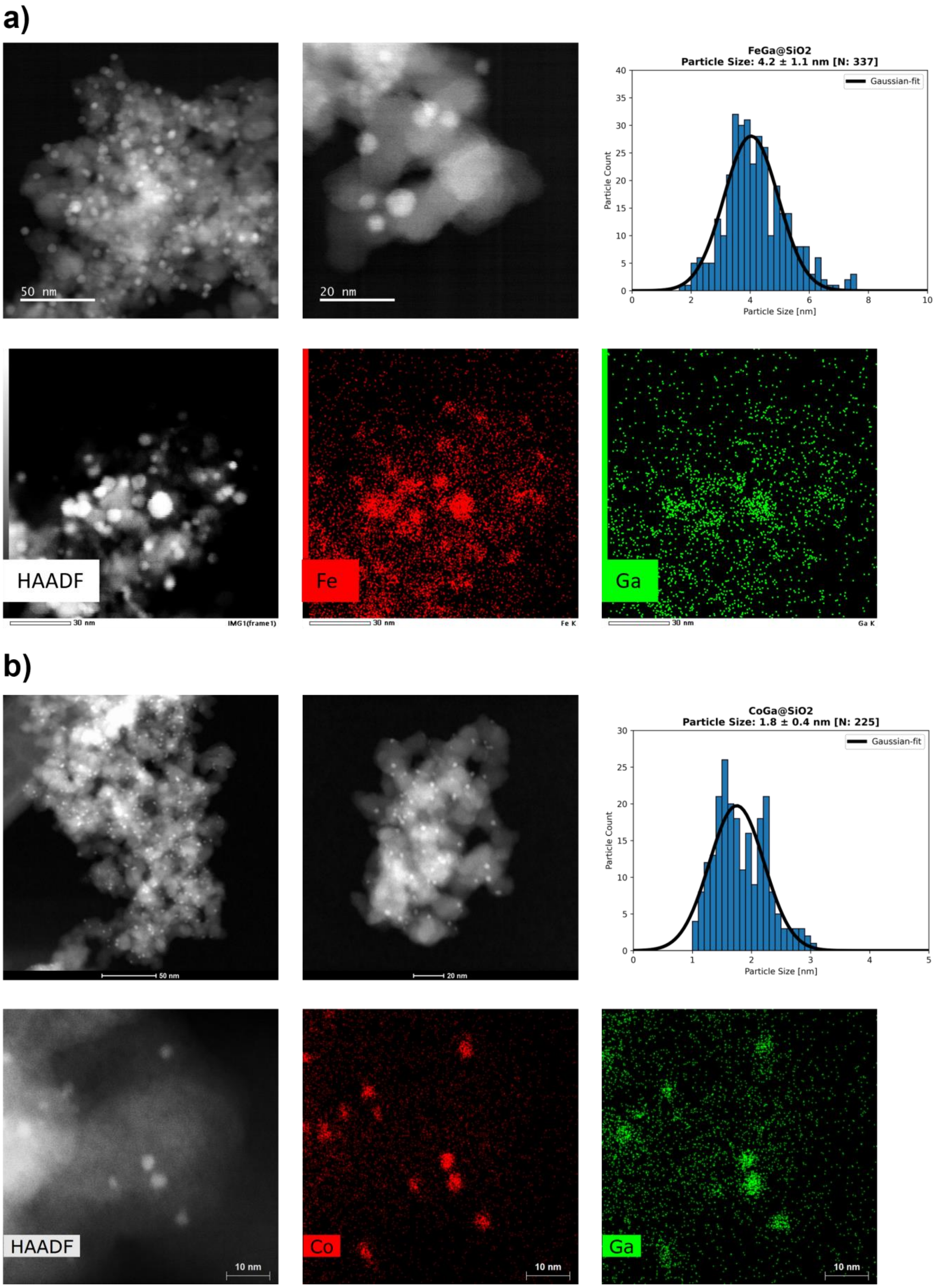

Figure S 4. HAADF-STEM and EDX Maps for $FeGa@SiO_2$ (a) and $CoGa@SiO_2$ (b).

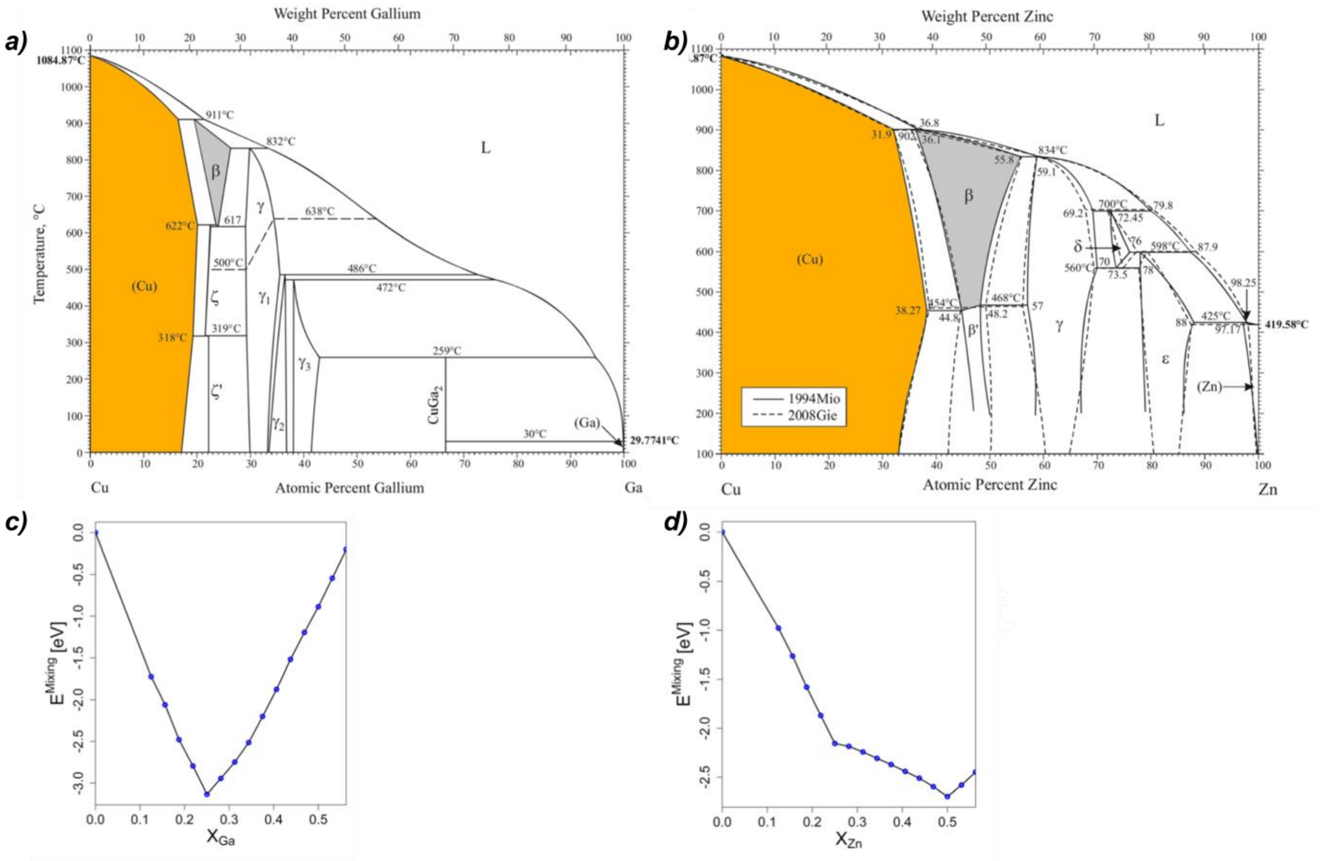

Figure S 5. Phase diagrams of the two bimetallic mixtures for the Cu-Ga (a) and Cu-Zn (b) systems. Figures taken and adapted from Okamoto[1]. For low concentrations of Ga/Zn, both structures crystallize in solid solutions with an fcc-structure of pure Cu modified by exchanging Cu with Ga/Zn (α-CuGa, α-CuZn coloured in orange). At higher concentrations, different crystal structures are obtained; Mixing energies for both fcc Cu-Ga (c) and fcc Cu-Zn (d) based on the molar fractions. The blue points indicate datapoints obtained from calculations of explicit bulk structures (figures adapted from Müller[2]).

References

(1) Okamoto, H. Supplemental Literature Review of Binary Phase Diagrams: Al-Nd, Al-Sm, Al-V, Bi-Yb, Ca-In, Ca-Sb, Cr-Nb, Cu-Ga, Ge-O, Pt-Sn, Re-Y, and Te-Yb. *Journal of Phase Equilibria and Diffusion* **2016**, *37* (3), 350-362.

(2) Müller, A.; Comas-Vives, A.; Copéret, C. Ga and Zn increase the oxygen affinity of Cu-based catalysts for the COx hydrogenation according to ab initio atomistic thermodynamics. *Chem. Sci.* **2022**, *13* (45), 13442-13458, 10.1039/D2SC03107H.
